# Effects of immune checkpoint inhibitors on the pulmonary circulation in lung cancer patients

**DOI:** 10.1002/ijc.70110

**Published:** 2025-09-05

**Authors:** Yao Xu, Qiuhong Zhang, Jie Gao, Shiyuan Yao, Chan Tian, Tuo He, Ming Zhang, Hu Shan, Jie Shi, Bo Yuan, Lei Wang, Xia Yang

**Affiliations:** ^1^ Department of Respiratory and Critical Care Medicine Second Affiliated Hospital of Xi'an Jiaotong University Xi'an China; ^2^ Department of Respiratory and Critical Care Medicine Gaoling District People's Hospital Xi'an China; ^3^ Department of Medical Imaging Second Affiliated Hospital of Xi'an Jiaotong University Xi'an China

**Keywords:** immune checkpoint inhibitors, lung cancer, prognosis, pulmonary circulation

## Abstract

Immune checkpoint inhibitors (ICIs) are effective anti‐tumor agents, but new immune‐related side effects (irAEs) are emerging. This retrospective cohort study investigated 461 lung cancer patients treated with ICIs over 2 years, analyzing changes in pulmonary artery diameter (PAD), aortic diameter (AoD), and the pulmonary artery/aortic diameter (PAD/AoD) ratio through chest computed tomography (CT) at baseline, 3 months, 6 months, 1 year, and 2 years post‐treatment. The PAD increased from 25.19 to 26.33 mm (*p* < .001) and the PAD/AoD ratio increased from 0.70 to 0.73 (*p* < .001) during ICI treatment over a 2‐year follow‐up period, as early as within the first 3 months. High‐sensitivity troponin I (hs‐TnI), α‐hydroxybutyrate dehydrogenase (HBDH), creatine kinase (CK), creatine kinase MB (CK‐MB), and coagulation indices exhibited significant changes (*p* < .05), while the cardiac ultrasound result remained unchanged. In subgroup analysis, the severe group demonstrated lower overall survival (OS) (43 months vs. 56 months) (*p* = .008) and progression‐free survival (PFS) (15 months vs. 20 months, *p* < .001) compared to the non‐severe group. Meanwhile, the severe PAD/AoD ratio progression served as independent predictors of prognosis in lung cancer patients receiving ICI treatment, but immune‐related pneumonia(CIP) did not significantly influence the PAD/AoD ratio progression (*p* > .999). Therefore, in lung cancer patients receiving ICIs, pulmonary vascular involvement can occur within the initial 3 months and needs to be monitored with chest CT and echocardiography.

## Graphical abstract

What's new?

Immune checkpoint inhibitors (ICIs) are effective against various cancers, but as more patients receive these therapies, more immune‐related adverse events (irAEs) emerge. Recent studies have suggested that ICIs are associated with an increase in pulmonary artery diameter and the ratio between pulmonary artery diameter and aortic diameter. Here, the authors show that these pulmonary symptoms may begin within 3 months of beginning ICI treatment and that a severe increase in the PAD/AoD ratio is an independent predictor of poor prognosis. This ratio should be carefully monitored in patients receiving ICI treatment.

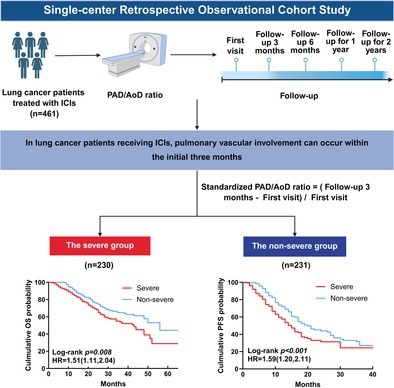

**Abbreviations**
ADK: adenocarcinoma
AoD: aortic diameter
APTT: activated partial thromboplastin
CEA: carcinoembryonic
CIP: immune‐related pneumonia
CK: creatine kinase
CK‐MB: creatine kinase MB
CR: complete response
CT: chest computed tomography
DCR: disease control rate
DDN: D‐dimer
FDP: fibrinogen degradation products
FIB: fibrinogen
FOXP3: forkhead lineage‐transcription factor 3
HBDH: α‐hydroxybutyrate
hs‐TnI: high‐sensitivity troponin
ICIs: immune checkpoint inhibitors
IL‐1β: interleukin‐1β
IL‐6: interleukin‐6
IL‐10: interleukin‐10
IL‐17: interleukin‐17
INR: international normalized ratio
irAEs: immune‐related adverse events
MPV: mean platelet volume
NSCLC: non‐small cell lung cancer
ORR: the objective response rate
PAD: pulmonary artery diameter
PAD/AoD ratio: pulmonary artery/aortic diameter ratio
PD: progressive disease
PDW: platelet distribution width
PFS: progression‐free survival
PGI2: prostacyclin
PH: pulmonary hypertension
PR: partial response
PT: prothrombin time
PTA: prothrombin activity
PTR: prothrombin time ratio
SCC: squamous carcinoma
SCLC: small‐cell lung cancer
SD: stable disease
Th: cell helper T cell
Treg: cell regulatory T cell
TT: thrombin
VTE: venous thromboembolism

## 1 INTRODUCTION

In the contemporary medical landscape, immune checkpoint inhibitors (ICIs) have emerged as a pivotal antitumor therapy, rapidly evolving into a prevalent treatment modality. This synergistic integration of ICIs with established therapeutic approaches—such as chemotherapy, radiotherapy, targeted therapy, and antiangiogenic therapy—has led to substantial advancements in life expectancy for numerous cancer patients. Nevertheless, immune‐related adverse events (irAEs) should not be ignored. The disruption of immune tolerance and the presentation of autoantigens can lead to irAEs that affect multiple systems throughout the body.^1^, ^2^ For lung cancer patients receiving immunotherapy, irAEs primarily impact the cardiac, pulmonary, gastrointestinal, endocrine, cutaneous, and nervous systems.^3^, ^4^ While most adverse effects are manageable, severe irAEs can occur, potentially leading to a significant reduction in patient survival.^5^ Studies indicate that the overall incidence of any grade of irAEs in patients with non‐small cell lung cancer (NSCLC) is approximately 30%, with severe cases reported at a rate of 6%.^6^ Interestingly, recent evidence suggests that certain irAEs may correlate with better prognostic outcomes, for instance, skin toxicity or endocrine toxicity is often predictive of improved outcomes.^7^, ^8^, ^9^ Nonetheless, it remains critical to acknowledge that severe adverse effects can still significantly impact a patient's expected survival.

Newly identified irAEs are emerging; recent literature has documented cases of pulmonary hypertension (PH) following ICI treatment, and precapillary pulmonary hypertension has been confirmed through a floating catheter in the right heart.^10^, ^11^, ^12^ Additionally, two relevant studies monitoring 59 and 24 lung cancer patients treated with ICIs have demonstrated that ICIs are associated with an increase in pulmonary artery diameter (PAD) and the pulmonary artery/aortic diameter (PAD/AoD) ratio, and some patients exhibited right heart dysfunction, which raised significant concerns within the clinical community.^13^, ^14^ Recent research suggested that the PAD/AoD ratio may be a prognostic marker for NSCLC patients. In addition, this study selected 100 NSCLC patients who received non‐ICI treatments and evaluated their PAD/AoD ratios before and after treatment to exclude the potential influence unrelated to immune therapy. The analysis revealed no statistically significant differences before and after therapy.^15^ Additionally, Fournel et al.^14^ demonstrated that conventional chemotherapy, unlike immunotherapy, does not induce changes in the PAD/AoD ratio. These findings imply that modifications in the PAD/AoD ratio may be specifically associated with immunotherapeutic interventions. Consequently, the potential for ICIs to induce pulmonary circulation dysfunction in lung cancer patients deserves further study. Simultaneously, whether the PAD/AoD, which is a measurable imaging marker in chest computed tomography (CT) images, can be a relevant predictor also merits further exploration.

Therefore, this paper aims to investigate whether ICIs lead to dysfunction of the pulmonary circulation system, to assess the utility of the PAD/AoD as a prognostic predictor, and to discuss related risk factors, aiming to provide a reliable basis for predicting ICI‐induced pulmonary circulation dysfunction in clinical management.

## 2 MATERIALS AND METHODS

### 2.1 Study design and patient selection

This study was a single‐center retrospective observational cohort investigation that included lung cancer patients treated with ICIs from January 1, 2019 to December 31, 2024, at the Second Affiliated Hospital of Xi'an Jiaotong University. The inclusion and exclusion criteria were as follows:

The inclusion criteria: (1) patients diagnosed with lung cancer according to the NCCN Clinical Practice Guidelines in Oncology^16^; (2) patients aged over 18 years; (3) at least two or more chest CTs; (4) patients treated with ICIs for more than 4 cycles; and (5) all signed informed consent. Exclusion criteria: (1) patients with incomplete or difficult‐to‐evaluate clinical or imaging data; (2) patients with other primary malignancies; (3) only one chest CT.

### 2.2 Clinical data collection

The collection of baseline data was conducted retrospectively from clinical cases of patients, encompassing essential clinical information such as age, gender, smoking history, body mass index, performance status, and a comprehensive history of previous diseases, including respiratory disease, hypertension, cardiac disease, autoimmune disease, and thromboembolism. Additional data included the pathological characteristics of the tumor, TNM stage, and the history of prior antitumor treatments, such as chemotherapy, radiotherapy, targeted therapy, the specific types of ICIs received, and so on. Laboratory data include routine blood tests, liver function, kidney function, blood lipids, thyroid function, markers of myocardial injury, cardiac enzymes, coagulation tests, and other relevant tests.

### 2.3 Imaging data acquisition

Imaging parameters were obtained from chest computed tomography (CT) and cardiac ultrasound at initial diagnosis, as well as at 3 months, 6 months, 1 year, and 2 years of follow‐up following the initiation of ICI treatment. Chest CT and cardiac ultrasound scans were conducted using consistent imaging parameters across all patients. The PAD was measured at the level of the pulmonary artery bifurcation in the axial plane, perpendicular to the direction of blood flow. The internal diameters of the PAD and aortic diameter (AoD) were measured at the same horizontal level, following the methodology described by Wells et al.^17^ Assessments were performed independently by two blinded observers, with each measurement taken three times. The PAD/AoD ratio was calculated by averaging the three indices.

### 2.4 Statistical analysis

Normal distribution measurements were expressed as mean ± standard deviation (M ± SD) and compared between groups using the paired‐samples *t* test; skewed distribution measurements were expressed as median (M) and quartiles (P25, P75). The Mann–Whitney *U* test or Wilcoxon's rank‐sum test was used to compare these skewed distribution measurements between groups. The Pearson *χ* ^2^ test and Fisher's exact probability method were used to compare counts, expressed as relative (percentage), between groups. All significant variables with *p*‐values less than .05 were analyzed for relevant independent predictors using multifactor Cox regression. It was considered that differences were statistically significant at *p* < .05 for all analyses.

### 2.5 Follow‐up and primary outcome

Follow‐up data were collected through clinical records, imaging assessments, and telephone follow‐up. All patients were followed up until 31 December 2024 or until patient death. The primary endpoint was overall survival (OS), defined as the duration from initiation of ICI treatment to death from any cause. Secondary endpoints include progression‐free survival (PFS). PFS is defined as the duration from the initiation of ICI treatment to disease progression or death in patients.

For patients who were lost to follow‐up, PFS was calculated using the date of the last imaging assessment as the cut‐off point; OS was calculated using survival status obtained through telephone follow‐up, with deceased patients counted as events and surviving patients counted as of the last follow‐up date. All deaths outside the hospital were not included as PFS events due to a lack of imaging confirmation of progression.

## 3 RESULTS

### 3.1 Patient characteristics

A total of 461 lung cancer patients treated with ICIs were enrolled in this retrospective cohort study. All patients had no pulmonary hypertension at the initial diagnosis. The follow‐up assessments were at 3 months (*n* = 461), 6 months (*n* = 327), 1 year (*n* = 217), and 2 years (*n* = 97). Each patient underwent a chest CT scan at each follow‐up interval. The cohort consisted of 72 (15.6%) females and 389 (84.4%) males, with 221 (47.9%) patients aged ≥65 years and 240 (52.1%) aged <65 years. Among the patients, 437 (94.6%) were treated with PD‐1 inhibitors while 25 (5.4%) received PD‐L1 inhibitors. And 284 (61.6%) patients underwent ≥6 cycles, while 177 (38.4%) patients received <6 cycles. The pathological classification revealed 204 (44.3%) cases of squamous carcinoma (SCC), 178 (38.6%) cases of adenocarcinoma (ADK), and 67 (14.5%) cases of small‐cell lung cancer (SCLC). During the treatment period, 42 (9.1%) patients developed deep venous thrombosis (DVT), while 17 (3.7%) patients experienced pulmonary embolism (PE). Additionally, 133 (28.9%) patients exhibited irAEs, which included 18 (3.9%) cases of immune‐related pneumonia (CIP). All the demographic characteristics of the cohort are summarized in Table S1, Supporting Information.

### 3.2 PAD/AoD ratio progression

Throughout the follow‐up period, the PAD increased from 25.19 (22.67, 27.86) mm to 26.33 (23.79, 28.34) mm (*p* < .01), and the PAD/AoD ratio increased from 0.70 (0.62, 0.76) to 0.73 (0.67, 0.77) (*p* < .01). Statistically significant differences in both PAD and the PAD/AoD ratio were observed at all follow‐up points compared to the initial diagnosis (*p* < .001). However, the change in AoD did not demonstrate statistical significance (*p* > .05) (Table S2). Among the 461 cases enrolled, 97 patients were selected who had undergone chest CT scans at each follow‐up visit. The result indicated that the changes in PAD and PAD/AoD ratio are statistically significant over the 2‐year follow‐up period (*p* < .01), whereas the changes in AoD are not (*p* = .493). Further comparisons revealed a statistically significant difference in PAD and PAD/AoD ratio from the first visit vs. 3 months, first visit vs. 6 months, first visit vs. 1 year, and first visit vs. 2 years (*p* < .001), but when comparing two adjacent follow‐up points, except for the comparison of the initial visit vs. 3 months (*p* < .001), no statistically significant differences were found between 3 months vs. 6 months, 6 months vs. 1 year, or 1 year vs. 2 years (*p* > .05) (Table S3).

### 3.3 Myocardial injury markers and cardiac ultrasound‐related indicators progression

A total of 69 patients were selected from the study population who had both myocardial injury markers and cardiac enzymes (all the demographic characteristics of the cohort are summarized in Table S6). The analytical result demonstrated statistically significant differences in high‐sensitivity troponin I (hs‐TnI), α‐hydroxybutyrate dehydrogenase (HBDH), creatine kinase (CK), and creatine kinase MB (CK‐MB) over the 2‐year follow‐up period (*p* < .05), while no significant differences were observed in the remaining markers (Table S4).

Furthermore, only nine patients underwent cardiac ultrasound during the follow‐up period. The results indicated that cardiac ultrasound‐related indicators did not yield statistically significant results throughout the follow‐up, which may be due to the small sample size.

### 3.4 Coagulation function changes

Seventy‐two patients with complete coagulation indices over a 2‐year follow‐up period demonstrated statistically significant changes in all coagulation parameters, including prothrombin time (PT), prothrombin time ratio (PTR), international normalized ratio (INR), prothrombin activity (PTA), activated partial thromboplastin time (APTT), fibrinogen (FIB), thrombin time (TT), fibrinogen degradation products (FDP), and D‐dimer (DDN) (*p* < .05). Furthermore, further comparisons revealed statistically significant differences between the initial visit vs. 3 months, and 1 year vs. 2 years. However, no significant differences were found between the other adjacent follow‐up points (Table S5).

### 3.5 Univariate and multifactorial analyses

Univariate and multivariate analyses were conducted on the basic clinical characteristics of the study population and their association with OS and PFS. The results demonstrated that, in the univariate analysis, the following factors were statistically significant predictors of OS: severe PAD/AoD ratio progression, age, smoking history, Eastern Cooperative Oncology Group (ECOG) performance status score (PS), histologic type, and stage. These variables were then incorporated into a multifactorial Cox regression model, and the results demonstrated that severe PAD/AoD ratio progression, age, PS score, histologic type, and stage were independent predictive factors for OS (Table 1).

**TABLE 1:** Univariate and multivariate Cox proportional hazards analyses for OS.

Parameter	Univariate analysis		Multivariate analysis	
	Hazard ratio (95% CI)	*p*‐value	Hazard ratio (95% CI)	*p*‐value
Groups				
Non‐severe group	Reference		Reference	
Severe group	1.51 (1.11, 2.04)	.**008**	1.46 (1.07, 1.98)	.**016**
Gender				
Male	Reference			
Female	0.77 (0.50, 1.20)	.244		
Age				
<65	Reference		Reference	
≥65	1.54 (1.13, 2.08)	.**006**	1.59 (1.17, 2.16)	.**003**
Smoking				
No	Reference			
Yes	1.39 (1.03, 1.88)	.**034**		
Body mass index				
<24	Reference			
≥24	0.71 (0.49, 1.04)	.082		
Performance status				
0–1	Reference		Reference	
2–4	2.79 (1.55, 5.03)	***<.001***	2.80 (1.50, 5.22)	.**001**
Respiratory disease				
No	Reference			
Yes	1.09 (0.69, 1.72)	.716		
Hypertension				
No	Reference			
Yes	0.79 (0.56, 1.12)	.188		
Diabetes				
No	Reference			
Yes	1.53 (1.00, 2.38)	.051		
Cardiovascular disease				
No	Reference			
Yes	1.11 (0.69, 1.79)	.671		
Autoimmune disease				
No	Reference			
Yes	3.96 (0.98, 16.01)	.053		
Thromboembolic history				
No	Reference			
Yes	1.71 (0.93, 3.17)	.086		
Deep venous thrombosis				
No	Reference			
Yes	1.36 (0.85,2.20)	.203		
Pulmonary embolism				
No	Reference			
Yes	1.47 (0.75, 2.88)	.260		
Histologic type of lung carcinomas		***<.001***		***<.001***
SCC	Reference		Reference	
ADK	1.04 (0.73, 1.47)	.837	1.19 (0.83, 1.71)	.344
SCLC	2.20 (1.48, 3.25)	***<.001***	2.19 (1.47, 3.28)	***<.001***
Other	0.59 (0.19, 1.87)	.369	0.48 (0.15, 1.55)	.221
Stages		***<.001***		.**005**
Stage I + II	Reference		Reference	
Stage III	2.82 (1.43, 5.56)	.**003**	2.62 (1.32, 5.20)	.**006**
Stage IV	3.55 (1.85, 6.79)	***<.001***	2.96 (1.54, 5.69)	.**001**
PD‐L1 (%)				
<1%	Reference			
≥1%	1.16 (0.59, 2.28)	.660		
Types of ICIs				
PD‐L1	Reference			
PD‐1	1.16 (0.54, 2.47)	.707		
Cycle of ICIs				
<6	Reference			
≥6	0.81 (0.59, 1.10)	.175		
Gene mutation				
No	Reference			
Yes	0.89 (0.60, 1.33)	.566		
Antiangiogenic inhibitors				
No	Reference			
Yes	0.94 (0.60, 1.47)	.774		
Chemotherapy, *n* (%)				
No	Reference			
Yes	0.75 (0.46, 1.24)	.264		
Thoracic radiotherapy, *n* (%)				
No	Reference			
Yes	0.83 (0.61, 1.14)	.250		
Therapeutic line, *n* (%)				
Non‐first‐line	Reference			
First‐line	0.77 (0.55, 1.10)	.148		
Therapeutic options		.674		
Immunotherapy	Reference			
Immunotherapy + other	0.74 (0.45, 1.23)	.246		
Immune‐related adverse events				
No	Reference			
Yes	1.20 (0.87, 1.65)	.256		
Neutrophil to lymphocyte ratio				
≤2	Reference			
>2	0.75 (0.52, 1.07)	.117		
Platelet to lymphocyte ratio				
≤150	Reference			
>150	0.86 (0.64, 1.16)	.329		

*Note*: Bold and italic values indicate *p* < 0.05 (statistically significant).

Abbreviations: PFS, progression‐free survival; CI, confidence interval; ICIs, immune checkpoint inhibitors; PD‐1, programmed cell death protein 1; PD‐L1, programmed cell death ligand 1.

In the univariate analysis of PFS, severe PAD/AoD ratio progression, thromboembolic history, deep venous thrombosis, type of ICI, therapeutic lines, and ICI treatment cycle were identified as potential influencing factors. Following the incorporation of these indicators into a multivariable Cox regression model, the analysis results indicated that all of the aforementioned factors functioned as independent predictors of PFS (Table 2).

**TABLE 2:** Univariate and multivariate Cox proportional hazards analyses for PFS.

Parameter	Univariate analysis		Multivariate analysis	
	Hazard ratio (95% CI)	*p*‐value	Hazard ratio (95% CI)	*p*‐value
Groups				
Non‐severe group	Reference		Reference	
Severe group	1.59 (1.20, 2.11)	***<.001***	1.44 (1.08, 1.92)	.**013**
Gender				
Male	Reference			
Female	0.92 (0.62, 1.34)	.648		
Age				
<65	Reference			
≥65	1.00 (0.76, 1.32)	.979		
Smoking				
No	Reference			
Yes	1.13 (0.86, 1.49)	.366		
Body mass index				
<24	Reference			
≥24	0.74 (0.53, 1.04)	.830		
Performance status				
0–1	Reference			
2–4	1.21 (0.61, 2.38)	.587		
Respiratory disease				
No	Reference			
Yes	1.15 (0.78, 1.71)	.476		
Hypertension				
No	Reference			
Yes	0.79 (0.58, 1.07)	.133		
Diabetes				
No	Reference			
Yes	0.82 (0.51, 1.32)	.417		
Cardiovascular disease				
No	Reference			
Yes	0.92 (0.58, 1.46)	.709		
Autoimmune disease				
No	Reference			
Yes	4.48 (0.62, 32.27)	.103		
Thromboembolic history				
No	Reference		Reference	
Yes	2.12 (1.24, 3.60)	.**005**	1.80 (1.06, 3.06)	.**031**
Deep venous thrombosis				
No	Reference		Reference	
Yes	1.55 (1.01, 2.37)	.**042**	1.63 (1.06, 2.51)	.**025**
Pulmonary embolism				
No	Reference			
Yes	1.72 (0.91, 3.26)	.091		
Histologic type of lung carcinomas				
SCC	Reference			
ADK	1.10 (0.82, 1.48)	.538		
SCLC	1.28 (0.84, 1.95)	.256		
Other	0.85 (0.37, 1.97)	.704		
Stages				
Stage I + II	Reference			
Stage III	1.22 (0.77, 1.93)	.407		
Stage IV	1.27 (0.83, 1.96)	.275		
PD‐L1 (%)				
<1%	Reference			
≥1%	0.83 (0.50, 1.37)	.456		
Types of ICIs				
PD‐L1	Reference		Reference	
PD‐1	0.52 (0.30, 0.92)	.**024**	0.46 (0.26, 0.81)	.**007**
Cycle of ICIs				
<6	Reference		Reference	
≥6	0.39 (0.29, 0.53)	***<.001***	0.39 (0.29, 0.52)	***<.001***
Gene mutation				
No	Reference			
Yes	1.02 (0.73, 1.41)	.921		
Antiangiogenic inhibitors				
No	Reference			
Yes	0.86 (0.59, 1.28)	.461		
Chemotherapy, *n* (%)				
No	Reference			
Yes	0.88 (0.56, 1.38)	.579		
Thoracic radiotherapy, *n* (%)				
No	Reference			
Yes	0.98 (0.73, 1.32)	.894		
Therapeutic line, *n* (%)				
Non‐first‐line	Reference		Reference	
First‐line	0.63 (0.47, 0.85)	.**002**	0.64 (0.48, 0.87)	.**004**
Therapeutic options		.064		
Immunotherapy	Reference			
Immunotherapy + other	1.63 (0.93, 2.86)	.090		
Immune‐related adverse events				
No	Reference			
Yes	1.17 (0.88, 1.55)	.294		
Neutrophil to lymphocyte ratio				
≤2	Reference			
>2	1.18 (0.81, 1.71)	.395		
Platelet to lymphocyte ratio				
≤150	Reference			
>150	0.98 (0.74, 1.29)	.864		

*Note*: Bold and italic values indicate *p* < 0.05 (statistically significant).

Abbreviations: PFS, progression‐free survival; CI, confidence interval; ICIs, immune checkpoint inhibitors; PD‐1, programmed cell death protein 1; PD‐L1, programmed cell death ligand 1.

In summary, the results of univariate and multivariate analyses of OS and PFS indicate that severe PAD/AoD ratio progression may serve as a potential prognostic marker for lung cancer patients receiving ICI therapy.

### 3.6 Subgroup analysis

Four‐hundred and sixty‐one patients were included in this study and were standardized using the following formula: standardized PAD/AoD ratio = (PAD/AoD at 3 months of follow‐up − PAD/AoD at initial diagnosis)/PAD/AoD at initial diagnosis. A cut‐off value of the median, 0.032, was established, dividing the cohort into a severe group (>0.032, *n* = 230) and a non‐severe group (≤0.032, *n* = 231).^14^, ^15^ The median is a widely used classification method for continuous variables, including clinical, laboratory, and imaging indicators.^18^, ^19^, ^20^ Analysis of the two groups revealed statistically significant differences in PAD, AoD, and the PAD/AoD ratio (*p* < .05). However, no significant differences were observed between the groups regarding cardiac ultrasound results, myocardial injury markers, or myocardial enzymes. Correlation analysis of the baseline clinical characteristics indicated statistically significant differences (*p* < .05) in basophil cell count, MPV, platelet distribution width (PDW), blood creatinine, cystatin C, lipoprotein (a), and absolute CD8+ cell counts; the remaining baseline characteristics did not show statistical significance (Table S7).

### 3.7 Survival analysis assessment

**FIGURE 1:**
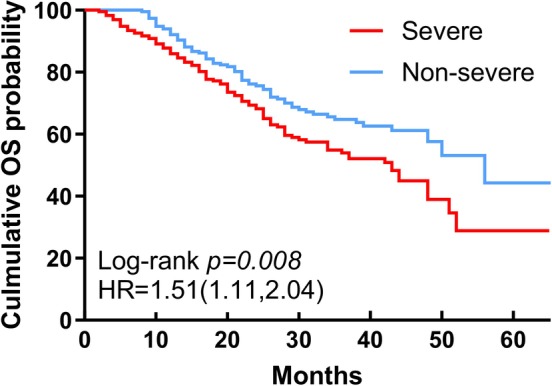
Kaplan–Meier curve of OS in non‐severe group (blue) and severe group (red). OS, overall survival.

**FIGURE 2:**
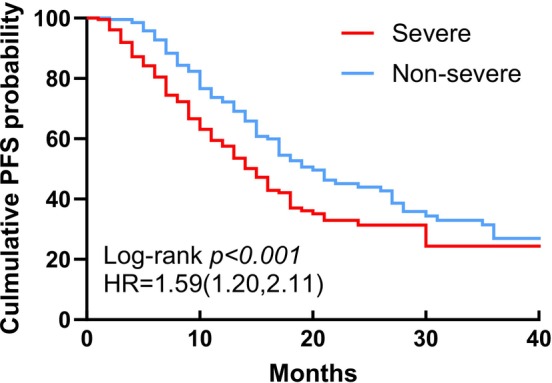
Kaplan–Meier curve of PFS in non‐severe group (blue) and severe group (red). PFS, progression‐free survival.

By the end of the follow‐up period, a total of 171 patients in the study cohort had reached OS, with 44 patients lost to follow‐up. Among these, 97 patients in the severe group and 74 patients in the non‐severe group had died. Kaplan–Meier analysis revealed a statistically significant difference in OS between the severe and non‐severe groups (mOS 43 months vs. 56 months) (*p* = .008) (Figure 1). Additionally, 210 patients had progressed to PD, with 44.2% of these patients belonging to the severe group. The Kaplan–Meier curve indicated a statistically significant difference in PFS between the two groups (mPFS 15 months vs. 20 months) (*p* < .001) (Figure 2).

**FIGURE 3:**
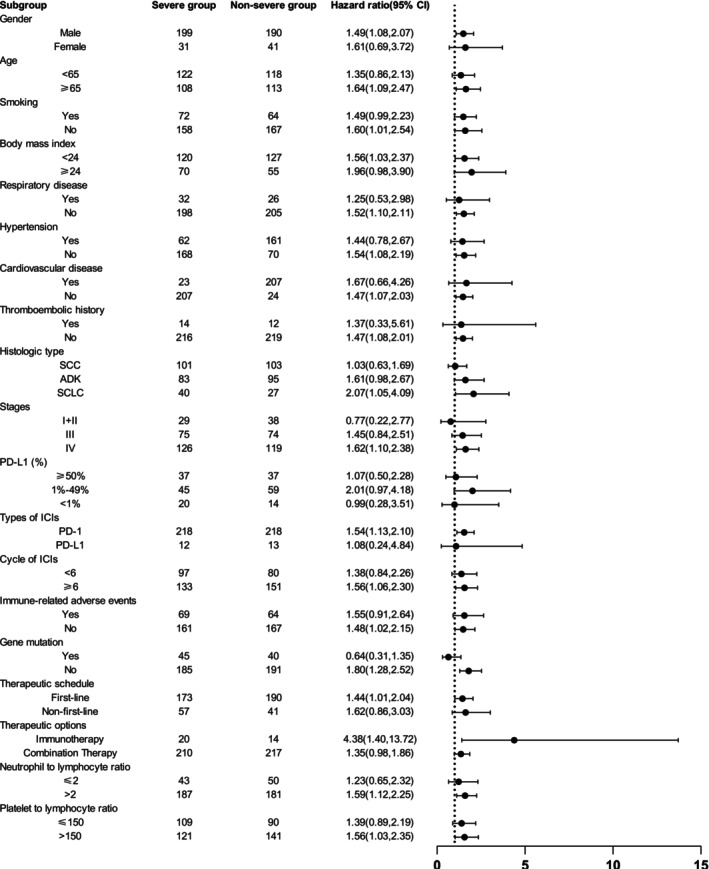
Subgroup analyses of overall survival (OS) between the severe and non‐severe groups. Hazard ratios were derived from the univariate Cox model for each subgroup. The dashed line indicates a hazard ratio of 1.

**FIGURE 4:**
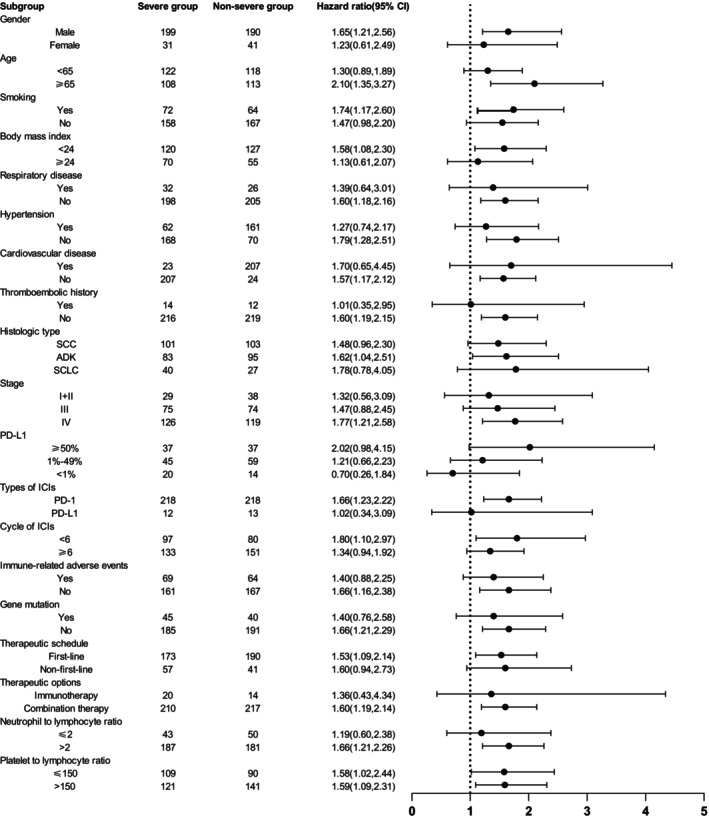
Subgroup analyses of progression‐free survival (PFS) between the severe and non‐severe groups. Hazard ratios were derived from the univariate Cox model for each subgroup. The dashed line indicates a hazard ratio of 1.

Subgroup analyses based on baseline clinical characteristics showed consistent trends. In each subgroup, except for the subgroup of gene mutation positivity, stage I + II and the PD‐L1 < 1%, the severe group had significantly shorter OS than the non‐severe group (Figure 3). In addition, in the PFS analysis, the severe group exhibited a significantly shorter PFS in each subgroup, except for the PD‐L1 < 1%. In the first‐line treatment subgroup, both OS and PFS were significantly reduced in the severe group compared to the non‐severe group (Figure 4). However, in subgroups with squamous cell carcinoma (SCC), stage III disease, and PD‐L1 expression level subgroups (>50% and 1–49%), although OS and PFS showed a trend toward shortening in the severe group, the differences were not statistically significant.

To further evaluate prognostic factors, a survival analysis was performed in a cohort of first‐line treatment patients with non‐squamous NSCLC without driver mutations. A total of 103 patients were included in this subgroup, with 45 in the severe group and 58 in the non‐severe group. The results demonstrated that, although the mOS in the non‐severe group had not yet been reached, there was a statistically significant difference in OS between the two groups (*p* = .002). Additionally, PFS was shorter in the severe group compared to the non‐severe group (mPFS 15 months vs. 19 months), though this difference did not reach statistical significance (*p* = .219).

Overall, except for a few specific subgroups, the severe group showed a trend toward shorter OS and PFS, though some subgroups did not show statistical significance.

### 3.8 Correlation between PAD/AoD ratio and immune‐related pneumonia

Eighteen out of 461 patients developed immune‐associated pneumonia (CIP), with 9 cases in both the severe and non‐severe groups. The analysis result indicated that the CIP did not significantly influence the progression of the PAD/AoD ratio (*p* > .999) (Figure S1). Furthermore, it demonstrated no significant effect on OS (*p* = .848) and PFS (*p* = .918) in either group (Figure S1).

## 4 DISCUSSION

Mylvaganam et al.^13^ found that ICIs were associated with right heart dysfunction and pulmonary vascular alterations. Based on this study, our investigation expanded the sample size to confirm whether ICIs can affect pulmonary circulation and their prognostic effect. ICIs can cause multiple cardiovascular toxicities, most notably myocarditis, pericarditis, conduction abnormalities, and vasculitis.^21^, ^22^ The study revealed that patients receiving ICIs were 11 times more likely to develop cardiovascular complications compared to those not receiving them, suggesting that ICIs may elevate the risk of cardiovascular disease in oncology patients, potentially leading to increased mortality.^23^ The underlying mechanisms may involve autoimmune dysregulation^24^ and T lymphocyte dysfunction.^25^, ^26^, ^27^, ^28^, ^29^

Immune dysfunction could play a significant role in PH pathogenesis.^30^ Many studies have focused on the balance of helper T (Th) and regulatory T (Treg) cells, with increasing evidence indicating that abnormalities in their number and function can contribute to the development of PH.^25^ In rat models, proliferation of Treg cells has been associated with elevated levels of prostacyclin (PGI2) and interleukin‐10 (IL‐10), both of which are known to reduce the risk of PH.^26^ Conversely, in PH patients, there is a notable elevation in Th cells, which can lead to increased production of pro‐inflammatory cytokines such as interleukin‐6 (IL‐6), interleukin‐1β (IL‐1β), and interleukin‐17 (IL‐17), and these cytokines are detrimental as they inhibit Treg cells' function and proliferation.^27^ Interestingly, while an increase in the proportion of Treg cells is observed in peripheral blood, a decrease in Treg cell numbers in lung tissues of PH patients suggests a reduction in their inhibitory function regarding PH development.^28^ Treg cells require continuous expression of the transcription factor forkhead lineage‐transcription factor 3 (FOXP3) for their stability and functionality, which could be influenced by the local tissue microenvironment, so ICIs may promote the development of PH by altering the tumor immune^29^ environment. The inhibition of PD‐1/PD‐L1 signaling could therefore increase the risk of PH via disruption in Treg cell pathways. In addition to Treg cells, the involvement of Th cells, particularly Th1, Th2, and Th17, in the development of PAH has been documented.^31^, ^32^ Th17 cells participate in both adaptive and innate immune responses through the secretion of IL‐17, promoting inflammatory processes within the body. Notably, the interaction between PD‐1 and PD‐L1 has been shown to limit Th17 polarization and contribute to the alleviation of PH in murine models. These findings suggest that ICI‐induced PH may partly result from enhanced Th17 immune polarization.^32^

Meanwhile, ICIs not only inhibit the PD‐1/PD‐L1 pathway, but they may also enhance pro‐atherosclerotic T‐cell responses, thereby accelerating atherosclerosis and contributing to cardiovascular toxicity.^33^ Additionally, ICIs can disrupt immune tolerance through several mechanisms, including T‐cell‐mediated cross‐reactivity between tumor cells and cardiomyocyte antigens, the promotion of autoantibody production, and an increase in pro‐inflammatory cytokines, leading to the development of cardiovascular toxicity.^21^, ^22^

This study aimed to compare changes in PAD and the PAD/AoD ratio following treatment with ICIs over a 2‐year follow‐up period. The results indicated an increase in the PAD/AoD ratio by 0.03 during this period, with further analysis revealing that this change primarily occurs within the first 3 months of treatment. Previous research has reported an increase in the PAD/AoD ratio from 0.75 to 0.78 following ICI therapy.^15^ Studies by Mylvaganam et al.^13^ and Palassin et al.^34^ demonstrated a significant increase in the PAD/AoD ratio at a median of 59 days post‐treatment initiation, with PH potentially induced at a median of 77 days, findings that are broadly consistent with our results. We also investigated changes in cardiac ultrasound parameters, markers of myocardial injury, and cardiac enzymes from the commencement of ICI therapy to the 2‐year follow‐up. The results revealed statistically significant differences in the levels of hs‐ThI, HBDH, CK, and CK‐MB, whereas no significant changes were observed in cardiac ultrasound indicators during the follow‐up period, which on the one hand may be attributed to the relatively small sample size of patients with cardiac ultrasound data; on the other hand, some studies suggest that cardiac ultrasound may be less sensitive in detecting PH.^35^ Therefore, for patients suspected of having PH, cardiac ultrasound in combination with cardiopulmonary exercise testing may be utilized for further diagnostic evaluation. Moreover, subgroup analysis indicated no differences in right heart function indices at initial diagnosis, further confirming that right heart circulatory system damage may occur during antitumor therapy with ICIs. This damage is primarily reflected in elevated markers of early myocardial injury and cardiac enzymes. These findings suggest that ICIs may significantly affect the pulmonary circulation within the first 3 months of treatment. Consequently, clinicians should regularly monitor changes in pulmonary artery diameter and the PAD/AoD ratio through chest CT and cardiac ultrasound, while also actively assessing myocardial injury markers and cardiac enzymes. Although our research found that the PAD/AoD ratio only increased by 0.03 within the 2‐year follow‐up, suggesting that it might not be a very significant change, the PAD/AoD ratio has potential clinical value as a prognostic biomarker for lung cancer patients with ICI therapy. Several studies have previously reported an upward trend in the PAD/AoD ratio among lung patients receiving ICI. However, there remains a lack of definitive evidence regarding whether the extent of change in this ratio meets established thresholds for clinical diagnosis or therapeutic intervention. Therefore, large‐scale, prospective studies are essential to further validate its clinical utility and establish standardized criteria. Moreover, compared to invasive right heart catheterization, the PAD/AoD ratio remains a relatively simple and non‐invasive index, with clear benefits for routine clinical monitoring. Its application is without obvious adverse effects on patients. In addition, the change in the PAD/AoD ratio underscores the importance of routine monitoring in patients receiving ICIs. An upward trend in this parameter may be an early warning sign of PAH, which is important for timely intervention and improving patient prognosis.

This study also discusses the impact of ICIs on coagulation. Analysis of coagulation indices during a 2‐year follow‐up after the initiation of ICIs revealed significant variation in the coagulation indices, but subgroup analysis indicated no significant differences in coagulation indices, venous thromboembolism (VTE), or pulmonary embolism between the severe and non‐severe groups, and these indices did not affect PFS. These findings suggest that ICIs may influence coagulation function in lung cancer patients, but their impact on PFS may not be significant. Moreover, the literature has reported venous thromboembolic events occurring in patients without a history of VTE after ICI treatment, most of which manifested as pulmonary embolism.^36^ In our study cohort, we also observed pulmonary embolism in patients without a history of VTE following ICI use, further supporting these findings. Although our study showed that both coagulation and venous thromboembolic events did not affect PFS, Gong et al.^37^ demonstrated that cancer patients treated with ICIs are at high risk of VTE and increase after starting an ICI, which may adversely affect their clinical course and prognosis.

The change of coagulation parameters often reflects an elevated risk of thrombotic events in clinical practice. Lung cancer patients are inherently in a hypercoagulable state due to various factors, which render them particularly susceptible to thrombosis. Therefore, routine monitoring of coagulation markers is essential, with D‐dimer and fibrinogen being the most informative indicators.^38^ These markers suggest ongoing coagulation and fibrinolytic activity, indicating an increased thrombotic risk. Vascular endothelial injury is a key initiator of coagulation activation, and pulmonary vascular endothelial cells are important regulatory factors of coagulation function. When there is a significant change in coagulation function, it also indicates that the pulmonary vascular endothelium may have suffered severe damage. The damage to pulmonary endothelial cells disrupts the balance between procoagulant and anticoagulant factors, resulting in increased vascular permeability, which can lead to pulmonary edema, heightened inflammation, and bleeding tendencies, ultimately worsening patient prognosis.^39^, ^40^ Therefore, coagulation indices need to be closely monitored after the initiation of anti‐cancer therapy with ICIs to prevent the development of pulmonary embolism.

We identified that severe progression of the PAD/AoD ratio was an independent predictor of prognosis in lung cancer patients by multifactorial Cox regression analysis. While the PAD alone proved to be an incomplete predictor of pulmonary hypertension, the presence of a mean PAD (mPAD) ≥29 mm yielded a positive predictive value of 97% for PH.^41^ Furthermore, PH was almost invariably observed in cases where the mPAD exceeded the AoD at the corresponding level on chest CT, suggesting that alterations in PAD serve as effective indicators of changes in pulmonary artery pressure and may signal the risk of PH.^42^ Consequently, a significant deterioration in the PAD/AoD ratio emerges as a risk predictor of PFS.

It has been hypothesized that CIP may affect the PAD/AoD ratio.^34^ Therefore, the study aims to further investigate the impact of CIP on the PAD/AoD ratio. The results indicated that CIP did not affect the PAD/AoD ratio, but it significantly shortened PFS in the severe group. Furthermore, Fournel et al.^14^ demonstrated that enlargement of the PAD was not associated with CIP, which aligns with the findings of this study. CIP is a rare irAE primarily resulting from the blockade of the PD‐1/PD‐L1 pathway, leading to the downregulation of protective Treg cells and Th2 cells.^43^ This disruption triggers immune dysregulation and results in lung injury. The manifestation of this injury is frequently observed as organic pneumonia, predominantly affecting both lungs, particularly the middle and lower lobes. It is often characterized by ground‐glass opacities and bronchiolar dilation observed on CT imaging, with less frequent involvement of the large pulmonary vessels.^44^

Previous studies have established that chemotherapy does not significantly alter the PAD/AoD ratio. In contrast, radiotherapy is another widely used anticancer modality, which primarily induces tumor cell death through ionizing radiation, but it can also cause various adverse effects, including pulmonary and cardiovascular toxicities. However, radiotherapy‐related cardiovascular toxicity is predominantly observed in patients with mediastinal Hodgkin lymphoma and breast cancer.^45^ In this study, 69.6% of the patients received thoracic radiotherapy. Due to the adverse effects of thoracic radiotherapy being mainly confined to the irradiated region, including early radiation pneumonitis and late fibrosis, the direct impact on the right cardiac function during the initial stages is generally limited. However, without appropriate intervention, progressive pulmonary and cardiovascular toxicity may lead to fibrotic changes, which can exert a contractile traction effect on the cardiac tissue, potentially impairing right ventricular function over time. Currently, there are no specific studies analyzing the effects of radiotherapy on the PAD/AoD ratio, underscoring the need for further research to validate these preliminary observations.

## 5 LIMITATIONS

This is a retrospective single‐center study, and the potential for selection bias is unavoidable. Although survival analyses were conducted, the absence of comprehensive follow‐up data on patient mortality may have resulted in incomplete outcomes for survival analyses. Furthermore, the relatively small number of patients with complete cardiac ultrasound during the 2‐year follow‐up period may impact the reliability of the results. While a thorough review of relevant literature was conducted and discussions regarding factors influencing the PAD/AoD ratio were included, the retrospective design precludes definitive establishment of a direct causal relationship between these variables. Meanwhile, the study lacks invasive right heart catheterization, which limits conducting an in‐depth analysis of the relationship between ICIs and pulmonary circulation/right heart dysfunction. This represents a significant limitation of the study. Future research should involve comprehensive, prospective, multicenter studies to further validate and expand upon these findings and to further explore the specific role of the PAD/AoD ratio in monitoring function in lung cancer patients.

## 6 CONCLUSIONS

During antitumor therapy with ICIs in patients with lung malignancies, there exists the possibility of pulmonary circulation injury, which is a risk factor for prognosis. Evidence for this risk is supported by the progression of the PAD/AoD ratio and the elevation of hs‐ThI and cardiac enzymes. Therefore, in clinical practice, the PAD/AoD ratio should be routinely assessed, and changes in hs‐ThI and cardiac enzymes should be monitored in patients receiving ICIs. This approach will facilitate the early identification of patients who benefit from ICI treatment versus those who may require alternative treatment strategies.

## AUTHOR CONTRIBUTIONS

**Yao Xu:** Formal analysis; investigation; project administration; writing – original draft. **Qiuhong Zhang:** Formal analysis; investigation; project administration; writing – original draft. **Jie Gao:** Investigation; visualization; writing – original draft. **Shiyuan Yao:** Investigation; visualization; writing – original draft. **Chan Tian:** Investigation; visualization; writing – original draft. **Tuo He:** Resources; writing – review and editing; supervision. **Ming Zhang:** Supervision; validation; writing – review and editing. **Hu Shan:** Writing – review and editing; validation; supervision. **Jie Shi:** Supervision; validation; writing – review and editing. **Bo Yuan:** Supervision; validation; writing – review and editing. **Lei Wang:** Conceptualization; methodology; data curation; funding acquisition; writing – review and editing. **Xia Yang:** Conceptualization; methodology; data curation; funding acquisition; writing – review and editing.

## CONFLICT OF INTEREST STATEMENT

The authors declare no conflicts of interest.

## ETHICS STATEMENT

This study has been approved by the Institutional Review Board of Xi'an Jiaotong University School of Medicine (approval number: 2025ER042). The ethics committee/institutional review board waived the requirement of written informed consent for participants because this study is retrospective. The study procedures were performed in compliance with Good Clinical Practice and the Declaration of Helsinki.

## Supporting information


**Data S1.** Supporting Information.

## Data Availability

All data generated or analyzed during this study are included in this published article and are available from the corresponding author upon reasonable request.
