# Supplementary material for: Effects of immune checkpoint inhibitors on the pulmonary circulation in lung cancer patients
Source: Int J Cancer. 2025 Sep 5;158(3):682–96. doi: 10.1002/ijc.70110 (PMC12670334; doi:10.1002/ijc.70110)

Supplementary Materials:

Effects of immune checkpoint inhibitors on the pulmonary circulation in lung cancer patients

Yao Xu, Qiuhong Zhang, Jie Gao, Shiyuan Yao, Chan Tian, Tuo He, Ming Zhang, Hu Shan, Jie Shi, Bo Yuan, Lei Wang , Xia Yang

Supplementary Tables:

Supplementary Table 1. Baseline characteristics

Supplementary Table 2. Computed tomography (n=461)

Supplementary Table 3. Computed tomography (n=97)

Supplementary Table 4. Myocardial markers (n=69)

Supplementary Table 5. Coagulation parameters (n=72)

Supplementary Table 6. Baseline characteristics for patients with cardiac markers (n=69)

Supplementary Table 7. Baseline characteristics of patients between the severe group and the non-severe group.

Supplementary Figures:

Supplementary Figure 1. Correlation analysis of CIP between non-severe group and severe group. CIP, immune-related pneumonia.

Supplementary Table 1. Baseline characteristics

Characteristics	Firsr visit n (%)	Follow-up for 2 years, n(%)	P value
Patient characteristics			
Patients, n	461	97	
Gender, n (%)			0.830
Male	389（84.4%）	81(83.5%)	
Female	72（15.6%）	16(16.5%)	
Age, n (%)			***0.012***
≥65	221（47.9%）	33(34.0%)	
<65	240（52.1%）	64(66.0%)	
Smoking history, n (%)			0.747
Never-smoker	236（51.3%）	54(55.7%)	
Current-smoker	136（29.6%）	26(26.8%)	
Former-smoker	88（19.1%）	17(16.2%)	
Performance status, n (%)			0.930
0-1	443（96.1%）	94(96.9%)	
2-4	18（3.9%）	3(3.1%)	
Body mass index , n (%)			0.925
≥24	125（33.6%）	28(34.1%)	
<24	247（66.4%）	54(65.9%)	
Respiratory disease, n (%)			0.534
Yes	58（12.6%）	10(10.3%)	
No	403（87.4%）	87(89.7%)	
Hypertension, n (%)			0.064
Yes	132（28.6%）	37(38.1%)	
No	329（71.4%）	60(61.9%)	
Diabetes, n (%)			0.391
Yes	48（10.4%）	13(13.4%)	
No	413（89.6%）	84(86.6%)	
Cardiovascular disease, n (%)			0.785
Yes	47（10.2%）	9(9.3%)	
No	414（89.8%）	88(90.7%)	
Autoimmune disease, n (%)			1.000
Yes	2（0.4%）	0(0.0%)	
No	459（99.6%）	97(100%)	
Thromboembolic history, n (%)			0.226

Yes	26（5.6%）	2(2.1%)	
No	435（94.4%）	95(97.9%)	
Deep venous thrombosis, n (%)			0.202
Yes	42(9.1%)	5(5.2%)	
No	419(90.9%)	92(94.8%)	
Pulmonary embolism, n (%)			0.303
Yes	17(3.7%)	1(1.0%)	
No	444(96.3%)	96(99.0%)	
Histologic type of lung carcinomas, n (%)			0.466
SCC	204（44.3%）	40(41.2%)	
ADK	178（38.6%）	45(46.4%)	
SCLC	67（14.5%）	10(10.3%)	
Other	12（2.6%）	2(2.1%)	
Stages, n (%)			0.917
Stage I	35（7.6%）	8(8.2%)	
Stage II	32（6.9%）	5(5.2%)	
Stage III	149（32.3%）	33(34.0%)	
Stage IV	245（53.2%）	51(52.6%)	
Diseased region, n (%)			0.942
Right lung	268（58.1%）	56(57.7%)	
Left lung	193（41.9%）	41(42.3%)	
PD-L1 (%), n (%)			0.954
≥50%	74（16.1%）	17(33.3%)	
1%-49%	104（22.6%）	25(49.0%)	
<1%	34（16%）	9(19.4%)	
Types of ICIs, n (%)			0.493
PD-1	437（94.6%）	90(92.8%)	
PD-L1	25（5.4%）	7(7.2%)	
Source of ICI, n (%)			0.154
Domestic	409（88.7%）	81(83.5%)	
Import	52（11.3%）	16(16.5%)	
Drug, n (%)			***0.026***
Sintilimab	150（32.5%）	23(23.7%)	
Tislelizumab	145（31.5%）	23(23.7%)	
Camrelizumab	41（8.9%）	13(13.4%)	
Other	125（27.1%）	38(39.2%)	
Cycle of ICIs, n(%)			***<0.001***
≥6	284（61.6%）	87(89.7%)	
<6	177（38.4%）	10(10.3%)	
Immune-related			0.361

adverse events, n (%)			
Thyroiditis	86（18.7%）	23(23.7%)	
Pneumonia	18（3.9%）	5(5.2%)	
Skin lesion	18（3.9%）	7(7.2%)	
Other	11（2.4%）	2(2.1%)	
No	328(71.1%)	60(61.9%)	
Gene mutation, n (%)			***0.036***
Yes	85（18.4%%）	27（27.8%）	
No	376（81.6%）	70（72.2%）	
Antiangiogenic inhibitors, n (%)			***0.009***
Yes	54（11.7%）	21(21.6%)	
No	407（88.3%）	76(78.4%)	
Chemotherapy, n (%)			0.736
Yes	423（91.8%）	90(92.8%)	
No	38（8.2%）	7(7.2%)	
Thoracic radiotherapy, n (%)			0.913
Yes	321（69.6%）	67(69.1%)	
No	140（30.4%）	30(30.9%)	
Therapeutic line, n (%)			0.668
1	363（78.7%）	75(77.3%)	
2	77（16.7%）	19(19.6%)	
3	21（4.6%）	3(3.1%)	
Therapeutic options, n (%)			0.103
Immunotherapy	34（7.4%）	9(9.3)	
Immunotherapy+ Chemotherapy	375（81.5%）	69(71.1%)	
Immunotherapy+ Chemotherapy+ Anti-angiogenesis	33（7.2%）	13(13.4%)	
Immunotherapy +other	18（3.9%）	6(6.2%)	

SCC, squamous-cell carcinoma; **ADK**, adenocarcinoma; **SCLC**, small-cell lung cancer; **ICIs**, immune checkpoint inhibitors; **PD-1**, programmed cell death protein 1; **PD-L1**, programmed cell death ligand 1.

Supplementary Table 2. Computed tomography (n=461)

	First Visit	Follow-up 3 months	Follow-up 6 months	Follow-up for 1 year	Follow-up for 2 years
PAD（mm）	25.19<br>(22.20,27.14)	25.98<br>(22.48,27.34)<br>(***<0.001***)*	25.99<br>(22.78,27.71)<br>(***<0.001***)*	26.14<br>(23.24,27.96)<br>(***<0.001***)*	26.33<br>(23.78,28.34)<br>(***<0.001***)*
AoD（mm）	36.24<br>(33.40,38.72)	36.19<br>(33.66,38.48)<br>(0.451)	36.07<br>(33.46,38.17)<br>(0.680)	36.25<br>(33.76,38.28)<br>(0.842)	36.28<br>(33.79,38.47)<br>(0.279)
PAD/AoD ratio	0.70<br>(0.66,0.77)	0.72<br>(0.66,0.78)<br>(***<0.001***)*	0.72<br>(0.67,0.78)<br>(***<0.001***)*	0.72<br>(0.66,0.78)<br>(<***0.001***)*	0.73<br>(0.67,0.78)<br>(<***0.001***)*

The results are shown as the medians and inter quartile ranges, with the corresponding p value below; **PAD**, pulmonary artery diameter; **AoD**, ascending aorta diameter; *Denotes significance at the p<0.05 level.

Supplementary Table 3. Computed tomography (n=97)

	first visit	Follow-up 3 months	Follow-up 6 months	Follow-up for 1 year	Follow-up for 2 years	P value
PAD（mm）	25.19 (22.67,27.86)	25.84 (23.30,27.76) ***(0.0007)******	26.22 (23.67,27.94) (***<0.001***)*	26.44 (23.41,27.96) (***<0.001***)*	26.33 (23.79,28.34) (***<0.001***)*	***<0.001***
AoD（mm）	36.42 (33.77,38.97)	36.01 (33.70,38.61) >0.999	36.26 (33.81,38.58) >0.999	36.42 (33.91,38.83) >0.999	36.28 (33.70,38.48) >0.999	0.493
PAD/AoD ratio	0.70 (0.62,0.76)	0.72 (0.65,0.77) (***<0.001***)*	0.72 (0.65,0.78) (***<0.001***)*	0.73 (0.65,0.77) (***<0.001***)*	0.73 (0.67,0.77) (***<0.001***)*	***<0.001***

The results are shown as the medians and inter quartile ranges, with the corresponding p-value below; **PAD**, pulmonary artery diameter; **AoD**, ascending aorta diameter; *Denotes significance at the *p<0.05* level.

Supplementary Table 4. Myocardial markers (n=69)

	First Visit	Follow-up 3 months	Follow-up 6 months	Follow-up for 1 year	Follow-up for 2 years	P value
BNP (pg/ml)	73.10 (49.30,75.60)	53.30 (21.50,158.90)	44.60 (32.20,70.20)	44.40 (28.60,103.40)	45.60 (29.00.342.20)	0.532
hs-TnI (ng/ml)	4.60 (3.00,8.00)	5.40 (3.70,13.20)	4.60 (3.20,10.80)	5.20 (2.70,9.60)	4.80 (4.20.9.30)	***0.003***
Mb(ng/ml)	16.50 (14.00,24.70)	24.70 (12.00,28.40)	27.20 (15.00,32.40)	24.50 (17.20,44.40)	28.80 (20.80,44.60)	0.108
HBDH (U/L)	128.00 (118.00,134.00)	149.00 (110.00,160.00)	131.00 (105.00,149.00)	120.00 (101.00,174.00)	124.00 (103.00,172.00)	***0.04***
LDH (U/L)	204.00 (179.00,239.00)	218.00 (156.00,285.00)	200.00 (165.00,246.00)	188.00 (161.00,267.00)	176.00 (152.00,265.00)	0.266
CK (U/L)	31.00 (28.00,111.00)	84.00 (37.00,139.00)	64.00 (41.00,210.00)	70.00 (44.00,115.00	58.00 (45.00,230.00)	***<0.001***
CK-MB (U/L)	12.00 (10.00,17.00)	13.00 (9.00,14.00)	10.00 (9.00,13.00)	11.00 (9.00,13.00)	13.00 (10.00,14.00)	***0.019***

The results are shown as the medians and inter quartile ranges, with the corresponding p-value; **BNP**, brain natriuretic peptide; **hs-TnI**, High-sensitivity troponin I; **Mb**, myoglobin; **HBDH**, α-hydroxybutyrate dehydrogenase; **LDH**, lactate dehydrogenase; **CK**, creatine kinase; **CK-MB**, creatine kinase MB.

Supplementary Table 5. Coagulation parameters (n=72)

	First Visit	Follow-up 3 months	Follow-up 6 months	Follow-up for 1 year	Follow-up for 2 years	P value
PT(s)	10.80 (10.40,11.40)	10.40 (9.70,11.00)	10.30 (9.90,10.90)	10.60 (10.20,11.00)	11.20 (10.50,11.60)	***<0.001***
PTR	0.97 (0.92,1.03)	0.95 (0.88,1.00)	0.94 (0.90,1.02)	0.97 (0.92,1.02)	1.01 (0.95,1.06)	***<0.001***
INR	0.97 (0.92,1.03)	0.95 (0.88,1.00)	0.94 (0.90,1.02)	0.96 (0.92,1.02)	1.01 (0.96,1.06)	***<0.001***
PTA	105.25 (93.30,115.85)	110.60 (95.70,127.70)	111.60 (97.00,128.50)	107.00 (93.90,114.60)	93.00 (86.10,107.70)	***<0.001***
APTT (s)	24.60 (22.20,27.40)	23.50 (21.50,26.70)	25.00 (22.70,27.00)	24.90 (22.50,26.70)	26.10 (22.80,29.40)	***0.016***
FIB (g/L)	5.99 (3.19,454.75)	3.37 (2.72,5.12)	3.23 (2.53,4.07)	3.17 (2.65,4.35)	3.48 (2.85,4.64)	***<0.001***
TT(s)	17.20 (16.63,17.70)	17.40 (16.80,18.20)	17.20 (16.70,17.90)	17.30 (16.50,18.20)	17.00 (15.80,17.50)	***<0.001***
FDP (mg/L)	1.91 (1.31,3.21)	1.71 (1.27,2.55)	1.69 (1.35,2.40)	1.83 (1.41,2.68)	2.25 (1.58,3.72)	***0.026***
DDN(mg/L)	650.00 (470.00,1345.00)	660.00 (450.00,930.00)	620.00 (470.00,820.00)	640.00 (530.00,940.00)	740.00 (580.00,1240.00)	***0.011***

The results are shown as the medians and inter quartile ranges, with the corresponding p-value; **PT**, Prothrombin time; **PTR**, Prothrombin time ratio; **INR**, International normalized ratio; **PTA**, Prothrombin activity; **APTT**, Activated partial thromboplastin time; **FIB**, Fibrinogen; **TT**, Thrombin time; **FDP**, Fibrinogen degradation products; **DDN**, D-dimer.

Supplementary Table 6. Baseline characteristics for patients with cardiac markers (n=69)

Characteristics		n(%)
Gender, n (%)		
	Male	56(81.2%)
	Female	13(18.8%)
Age, n (%)		
	≥65	28(40.6%)
	<65	41(59.4%)
Smoking history, n (%)		
	Never-smoker	34(49.3%)
	Current-smoker	23(33.3%)
	Former-smoker	12(17.4%)
Performance status, n (%)		
	0-1	67(97.1%)
	2-4	2（2.9%)）
Body mass index , n (%)		
	≥24	27(39.1%)
	<24	36(52.2%)
Respiratory disease, n (%)		
	Yes	8(11.6%)
	No	61(88.4%)
Hypertension, n (%)		
	Yes	27(39.1%)
	No	42(60.9%)
Diabetes, n (%)		
	Yes	11(15.9%)
	No	58(84.1%)
Cardiovascular disease, n (%)		
	Yes	6(8.7%)
	No	63(91.3%)
Autoimmune disease, n (%)		
	Yes	0(0.0%)
	No	69(100.0%)
Thromboembolic history, n (%)		
	Yes	0(0.0%)
	No	69(100.0%)
Histologic type of lung carcinomas, n (%)		
	SCC	33(47.8%)
	ADK	26(37.7%)
	SCLC	9(13.0%)
	Other	1(1.4%)
Stages, n (%)		
	Stage I	2(2.9%)

Stage II	4(5.8%)
Stage III	26(37.7%)
Stage IV	37(53.6%)
Diseased region, n (%)	
Right lung	37(53.6%)
Left lung	32(46.4%)
PD-L1 (%), n (%)	
≥50%	11(15.9%)
1%-49%	20(29.0%)
<1%	5(7.2%)
Types of ICIs, n (%)	
PD-1	62(89.9%)
PD-L1	7(10.1%)
Source of ICI, n (%)	
Domestic	57(82.6%)
Import	12(17.4%)
Drug, n (%)	
Sintilimab	15(21.7%)
Tislelizumab	18(26.1%)
Camrelizumab	7(10.1%)
Other	29(42.0%)
Cycle of ICIs, n(%)	
≥6	64(92.8%)
<6	5(7.2%)
Immune-related adverse events, n (%)	
Yes	37(53.6%)
No	32(46.4%)
Gene mutation, n (%)	
Yes	12(17.4%)
No	57(82.6%)
Antiangiogenic inhibitors, n (%)	
Yes	19(27.5%)
No	50(72.5%)
Chemotherapy, n (%)	
Yes	63(91.3%)
No	6(8.7%)
Thoracic radiotherapy, n (%)	
Yes	44(63.8%)
No	25(36.2%)
Therapeutic line, n (%)	
1	54(78.3%)
2	9(13.0%)
3	6(8.7%)
Therapeutic options, n (%)	

Immunotherapy	8(11.6%)
Immunotherapy+ Chemotherapy	47(68.1%)
Immunotherapy+ Chemotherapy+ Anti-angiogenesis	9(13.0%)
Immunotherapy +other	5(7.2%)

SCC, squamous-cell carcinoma; **ADK**, adenocarcinoma; **SCLC**, small-cell lung cancer; **ICIs**, immune checkpoint inhibitors; **PD-1**, programmed cell death protein 1; **PD-L1**, programmed cell death ligand 1.

Supplementary Table 7. Baseline characteristics of patients between the severe group and the non-severe group.

Characteristics	Severe group	Non-severe group	P value
Patients	230	231	
Gender, n (%)			0.207
Male	199(86.5%)	190(82.3%)	
Female	31(13.5%)	41(17.7%)	
Age, n(%)			0.673
<65	122(50.8%)	118(51.1%)	
≥65	108(47.0%)	113(48.9%)	
Smoking, n (%)	72(31.1%)	64(27.7%)	0.397
Body mass index, n (%)			0.176
<24	120(63.2%)	127(69.8%)	
≥24	70(36.8%)	55(30.2%)	
Performance status, n (%)			0.637
0-1	222(96.5%)	221(95.7)	
2-4	8(3.5%)	10(4.3%)	
Respiratory disease, n(%)	32(13.9%)	26(11.3%)	0.390
Hypertension, n (%)	62(27%)	70(30.3%)	0.427
Diabetes, n (%)	22(9.6%)	26(11.3%)	0.552
Cardiovascular disease, n (%)	23(10.0%)	24(10.4%)	0.890
Autoimmune disease, n (%)	0(0%)	2(0.9%)	0.096
Thromboembolic history, n (%)	14(6.1%)	12(5.2%)	0.678
Deep venous thrombosis, n (%)	23(10.0%)	19(8.2%)	0.508
Pulmonary embolism, n (%)	8(3.5%)	9(3.9%)	0.812
Diseased region, n (%)			0.281
Right lung	128(55.7%)	140(60.6%)	
Left lung	102(44.3%)	91(39.4%)	
Histologic type of lung carcinomas, n (%)			0.341
SCC	101(43.9%)	103(44.6%)	
ADK	83(36.1%)	95(41.1%)	
SCLC	40(17.4%)	27(11.7%)	
Other	6(2.6%)	6(2.6%)	
Stages, n (%)			0.493
Stage I+II	29(12.6%)	38(16.5%)	
Stage III	75(32.6%)	74(32.0%)	
Stage IV	126(54.8%)	119(48.6%)	
PD-L1 (%), n (%)			0.266
≥50%	37(36.3%)	37(33.7%)	
1%-49%	45(44.1%)	59(53.6%)	
<1%	20(19.6%)	14(12.7%)	
Types of ICIs, n (%)			0.846
PD-1	218(94.8%)	218(94.4%)	

PD-L1	12(5.2%)	13(5.6%)	
Drug, n (%)			0.119
Sintilimab	77(33.5%)	73(31.7%)	
Tislelizumab	65(28.3%)	80(34.6%)	
Camrelizumab	27(11.7%)	14(6.0%)	
Other	61(26.5%)	64(27.7%)	
Cycle of ICIs, n(%)			0.096
<6	97(42.2%)	80(34.6%)	
≥6	133(57.8%)	151(65.4%)	
Source of ICI, n (%)			0.974
Domestic	205(89.1%)	204(88.3%)	
Import	25(10.9%)	27(11.7%)	
Gene mutation, n (%)			0.534
Yes	45(19.6%)	40(17.3%)	
No	185(80.4%)	191(82.7%)	
Antiangiogenic inhibitors, n (%)			0.152
Yes	22(9.6%)	32(13.9%)	
No	208(90.4%)	199(86.1%)	
Chemotherapy, n (%)			0.171
Yes	207(90.0%)	216(93.5%)	
No	23(10.0%)	15(6.5%)	
Thoracic radiotherapy, n (%)			0.564
Yes	163(70.9%)	158(68.4%)	
No	67(29.1%)	73(31.6%)	
Therapeutic line, n (%)			0.065
First-line	173(75.2%)	190(82.3%)	
Non-first-line	57(24.8%)	41(17.7%)	
Therapeutic options			0.285
Immunotherapy	20(8.7%)	14(6.1%)	
Combination therapy	210(91.3%)	217(93.9%)	
Immune-related adverse events, n (%)	69(30%)	64(27.7%)	0.587
Immune-related pneumonia, n (%)			>0.999
Yes	9(3.9%)	9(3.9%0	
No	221(96.1%)	222(96.1%)	
Neutrophil to lymphocyte ratio, n (%)			0.430
≤2	43(18.7%)	50(21.6%)	
>2	187(81.3%)	181(78.4%)	
Platelet to lymphocyte ratio, n (%)			0.068
≤150	109(47.4%)	90(39.0%)	
>150	121(52.6%)	141(61.0%)	
Hemoglobin	125.00(115.50,133.50)	125.00(115.00,136.00)	0.473
Basophil Cell Count	0.02(0.02,0.04)	0.03(0.02,0.05)	***0.043***
Platelet distribution width	11.00(9.70,12.55)	11.90(10.10,13.15)	***0.015***
Mean platelet volume	10.50(9.60,11.20)	10.70(9.80,11.45)	***0.020***

Glomerular filtration rate	109.18(99.44,129.84)	111.87(101.52,133.01)	0.194
Serum creatinine	57.49(46.92,65.90)	51.93(45.23,62.44)	***0.005***
Blood urea nitrogen	4.80(3.90,5.80)	4.66(3.80,5.60)	0.396
Cystatin C	1.02(0.88,1.16)	0.96(0.85,1.10)	***0.016***
Albumin	37.40(34.00,40.50)	36.80(33.70,39.78)	0.110
High-density lipoprotein	1.13(0.29)	1.11(0.30)	0.442
Low-density lipoprotein	2.15(1.40,2.86)	2.29(0.40,2.89)	0.255
Very low-density lipoprotein	0.42(0.24)	0.43(0.23)	0.883
Lipoprotein a	15.50(8.50,36.90)	24.35(16.53,34.83)	***0.024***
Total bilirubin	10.80(8.60,13.50)	10.55(8.15,13.27)	0.471
Direct bilirubin	2.49(2.00,3.23)	2.57(1.94,3.38)	0.740
Indirect bilirubin	8.30(6.30,10.40)	7.78(5.98,10.22)	0.274
Absolute CD8+ cell counts	363.0(136.4)	489.4(172.7)	***0.049***
Brain natriuretic peptide	51.00(24.10,72.20)	45.40(20.10,63.05)	0.422
Troponin I	3.70(2.55,6.50)	3.90(2.53,4.93)	0.341
Myoglobin	19.80(15.00,26.90)	17.60(14.70,26.20)	0.082
Creatine kinase	51.00(32.00,80.00)	50.50(34.25,68.25)	0.831
Creatine kinase-MB	11.00(9.00,17.00)	12.50(11.00,16.50)	0.053
Thyroid Stimulating Hormone	2.12(1.18,2.88)	2.66(1.86,3.51)	0.587
Thyroid peroxidase antibody	11.87(10.10,17.48)	11.60(9.07,19.19)	0.673
Thyroglobulin antibody	17.22(14.30,20.00)	16.30(13.89,19.35)	0.618
Carcinoembryonic antigen	4.29(2.09,13.75)	3.83(2.08,8.95)	0.743
Neuron specific enolase	17.90(13.60,26.20)	16.60(13.35,18.73)	0.859
Gastrin-releasing peptide precursor	32.40(24.35,44.10)	28.05(21.73,40.33)	0.065
Prothrombin time	11.08(0.85)	10.62(0.74)	0.781
Prothrombin activity	98.66(17.61)	103.08(9.75)	0.870
Activated partial thromboplastin time	26.44(2.87)	28.96(4.82)	0.341
Fibrinogen	4.91(4.38,7.18)	4.31(2.72,5.21)	0.404
Fibrinogen degradation product	4.05(2.59)	3.33(2.02)	0.466
D-Dimer	830.00(617.50,1702.50)	970.00(660.00,2490.00)	0.880
Right ventricular transverse diameter	24.00(22.50,24.00)	24.00(23.00,24.75)	0.818
Right atrial transverse diameter	30.00(29.00,32.00)	31.00(28.00,32.00)	0.889
Interventricular septal thickness	10.00(10.00,10.00)	10.00(10.00,10.00)	0.124
Interventricular septum amplitude	9.00(8.00,9.000	8.00(8.00,8.75)	0.476
Left ventricular ejection fraction	0.66(0.64,0.69)	0.65(0.61,0.70	0.809
Maximum tricuspid regurgitation velocity	242.00(221.00,280.00)	245.00(220.75,274.50)	0.925

Abbreviations: **SCC**, squamous-cell carcinoma; **ADK**, adenocarcinoma; **SCLC**, small-cell lung cancer; **ICIs**, immune checkpoint inhibitors; **PD-1**, programmed cell death protein 1; **PD-L1**, programmed cell death ligand 1.

Supplementary Figure 1. Correlation analysis of CIP between non-severe group and severe group. CIP, immune-related pneumonia.

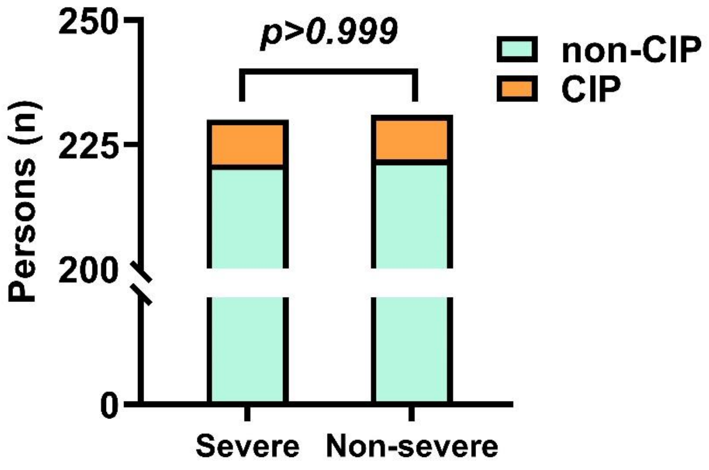

Supplement: Supplementary file 1 — Data S1. Supporting Information. [file IJC-158-682-s001.pdf]
